# “The Diseases of Society.”—Letter from the Author

**Published:** 1905-10

**Authors:** G. Frank Lydston

**Affiliations:** Chicago


### CORRESPONDENCE.

### "The Diseases of Society."—Letter from the Author.

*Editor Buffalo Medical Journal:*

SIR—I have just finished reading your exhaustive review of my recent book, "The Diseases of Society." I wish to express my sincere appreciation of the masterly manner in which your reviewer, "N. W. W.," presents the merits and demerits of the book. Frankly, his review is the most thorough of any which I have seen. It is a pleasure to read a review of one's work, which shows plainly that the reviewer has read the book intelligently and thoroughly. In general, the impression which his review made upon me was that he could have handled the subject in a much better way. Candidly, he rather than I should have written the book. I congratulate him upon his keen scent for the weak spots in my work. He has picked out the very things which I myself would have struck at had I been a disinterested reviewer. Probably because I was not perfectly lucid in expression, or was intentionally radical for the purpose of making the profession and public "sit up and pay attention," you do not in some respects interpret correctly the position which I intended to assume on some of the points. I note with considerable interest your comment on "the legal murder of two unfortunate youths," etc. I cannot believe that the murder which I described can justly be classed as premeditated, despite the manner in which the murderer procured the weapon. Premeditation on the part of a drunken and infuriated individual is something of which I cannot form a clear conception.

The point that I desired to make in connection with the case, and which possibly you overlooked—namely, that one of the men executed must of necessity have been an innocent party to the blow which destroyed the life of the victim. I regret very much your inference that I am defending anarchy. That was in no wise my intention. So far as philosophic anarchy is concerned, I have no defense to make for it, for it needs none. Philosophic anarchy is the *ultima thule* of idealistic individualism. Your true anarchist, of which Prince Krapotkin is a shining exemplar, is a dreamer who discounts the things which are, from his idealistic conception of what they ought to be. Given a human nature ideal, and the individualist would have none to gainsay the cogency of his arguments. Indeed, there would be no room for argument. It seems to me that the emphasis which I put upon the anarchistic trend of our social system in general, and the "ungloved" manner in which I handled social disturbers and corruptionists of all classes, should disabuse your mind of the idea that I am a sympathiser with "every day anarchy" on principle. I wish you would read carefully the chapter upon anarchy again. I thing that you will thereby form a different opinion of my attitude of mind. I, of course, recognise the difficulty of presenting my views clearly in a single chapter on a subject of such mighty social importance. The thing I wished to do especially, was to call attention to the tendency of our social system to draw a line of demarkation between such destructivists as those paranoiacs who were executed in Chicago, and such people as under the guise of trades unionists have been making Chicago a by-word and a reproach throughout the world for some months past. I also aimed to convey the idea that it were well to lay less stress upon the society disturbing tendencies of paranoiacs, and more upon the violence and corruption exhibited by supposedly respectable social elements, whether they represent the capitalistic or labor classes, whether they be politicians or exponents of high finance.

One disadvantage under which you labor in discussing the Haymarket anarchists is your lack of personal acquaintance with the men. You would be surprised, could you have known Albert Parsons and Spies, for example, at the intelligence and intellectual qualifications in general of these men. Not that they were any the less dangerous,—but let us be fair.

I wish, moreover, that you could have been in touch with the unfairness and hysteria which characterised the attitude of press, public and police toward the anarchists. At the time they were executed I was much more virulent in my condemnation of them than you are. I recall that several unbiased friends of mine, thinkers all, some of whom were eminent lawyers, told me that I would see the day when I would look at the legal aspect of the trial and execution in an entirely different light. Their prophesy case true many years later. I do not deny that society is better off without those men and their ilk, but that has no bearing upon the legality or illegality of the trial and execution. If I had my way about it, all such individuals would be put beyond the power to do harm to society, but it should not be done upon illogical grounds, nor in accordance with the principle of revenge. Nothing that anyone could say would change my opinion of the soundness and justice of Governor Altgeld's reasons for pardoning the anarchists who were imprisoned. I do not believe that a single flaw can be picked in his defense of his own position from a legal or moral standpoint. As to the question of social expediency and hysterical clamor for social revenge, it has no bearing upon the late Mr. Altgeld's attitude. Primarily prejudiced against him, I came to revere him as a philosopher and a conscientious humanitarian, who was fully fifty years in advance of his time.

With regard to Ling, there is no question in my mind or that of expert alienists who studied his case that he was insane. Had he not blown his head off, a new trial would have been obtained for all of the anarchists and I doubt very much whether, had the retrial been ordered, any of them would have been executed. I would infer from your criticisms that a "poor, fear-bitten creature, cowering under the outspread wings of death," could not be insane. "The stoicism of a martyr or the blind courage of a fanatic" are neither of them necessary to prove insanity. Cunning is characteristic of the insane. Ling's self-destruction was inspired by a mixture of cunning and desperate disregard for consequences to himself. To cheat the capitalistic classes out of the pleasure of destroying him upon the gallows was the mainspring of his mad suicidal act.

With regard to Czolgosz and Guiteau, and Prendergast, the murderer of Mayor Harrison, of Chicago, it would be very difficult to convince me that any of these men would have been executed had they murdered ordinary citizens. It by no means follows from this that I do not admit that the world is better off without them, and that they should have been removed from the society in which they were unequivocally dangerous elements. That, however, they should have been removed in the manner and form in which they were is to my mind open to serious argument. Frankly, while I do not believe in capital punishment, or in any form of social revenge, I should be heartily in accord with a provision for blotting from existence such dangerous social elements. The insane murderer rather than many of the sane is the one whose life I should destroy,—not for revenge, nor for punishment, but on the same principle that a farmer destroys the thistles in his cornfield.

Permit me to thank you again for your comprehensive review of a book which of necessity cannot be perfect or impregnable throughout, but which I hope may fulfil the promise which you have expressed for it.

G. Frank Lydston.

Chicago, August 4, 1905.

